# Light, Sleep, and Circadian Rhythms: Together Again

**DOI:** 10.1371/journal.pbio.1000145

**Published:** 2009-06-23

**Authors:** Derk-Jan Dijk, Simon N. Archer

**Affiliations:** Surrey Sleep Research Centre, Faculty of Health and Medical Sciences, University of Surrey, Guildford, United Kingdom

The 24-hour (h) light–dark (LD) cycle is a fundamental characteristic of Earth's environment and so its powerful influence on the behaviour and physiology of animals and humans that evolved on this planet is not surprising. In addition to influencing the perception of visual images, light coordinates the temporal rhythms of physiology and behaviour by sending signals to structures in the brain that contain the central circadian clock. These signals are mediated in part by melanopsin, a photopigment found in the retina. Light affects the brain through these nonvisual pathways, and scientists have recently begun to realize just how pervasive these nonvisual effects are. Mounting evidence supports the view that the effects of light on sleep and brain activity during wakefulness, as well as the duration of sleep and the homeostatic response to sleep loss, depend on both melanopsin and circadian time.

## Light and Entrainment of Circadian Rhythms

**Figure 1:**
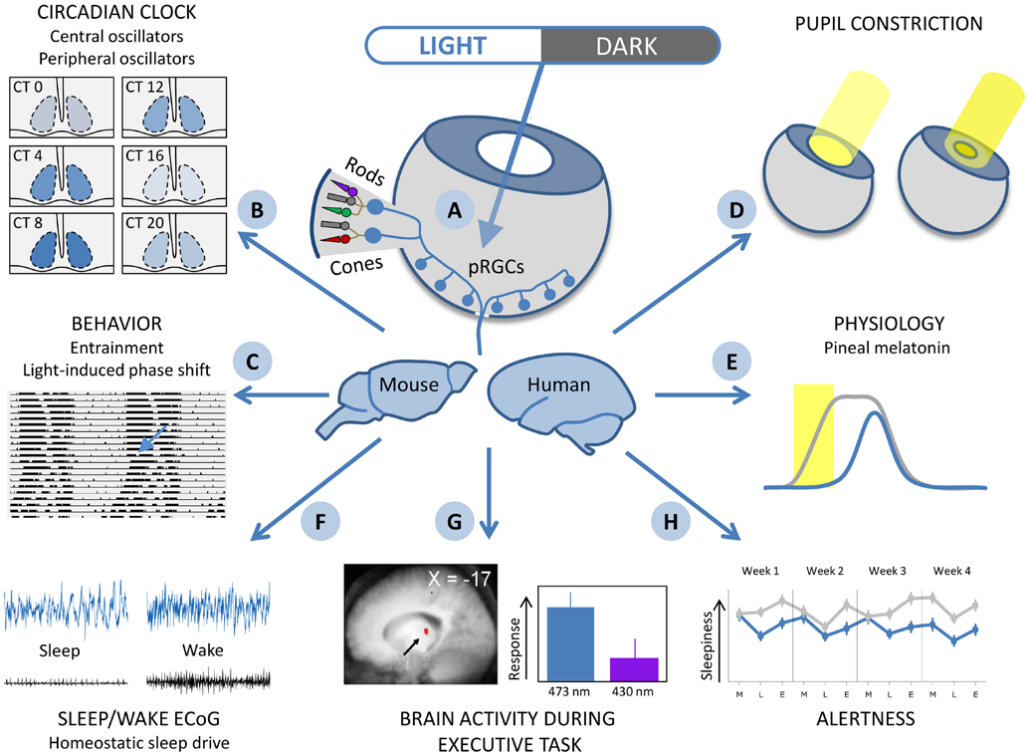
Summary of pervasive effects of light. A diffuse network of photosensitive retinal ganglion cells (pRGCs), which also receive input from rods and cones, are maximally sensitive to blue light between 470 and 480 nm (A). These cells have direct connections to the central circadian oscillator in the SCN where depending on the time of day (circadian time, CT) light induces changes in gene expression (B). pRGCs also mediate the synchronisation to LD cycles of locomotor activity, and light-induced phase shifts (C). pRGC connections to the olivary pretectal nucleus mediate light-sensitive pupil constriction (D), and indirect input via the SCN regulates the light-sensitive suppression of melatonin production in the pineal (E). The pRGC network has direct connections to sleep regulatory structures such as the VLPO and thereby modulates sleep and the ECoG during wakefulness (F). Blue light can modify brain responses to an executive task, as measured using fMRI (G) (figure adapted from [33], with permission), and can improve alertness (H) during the morning, lunch time, and early evening (figure based on data published in [32]).

In humans and other diurnal animals, most behavioural activity occurs during the day, whereas in nocturnal animals, such as mice, most activities are confined to the dark phase. Even in the absence of an LD cycle, the rest–activity rhythm persists with a periodicity of approximately 24 h, instead of redistributing across the 24-h day. More than 35 years ago, it was discovered that circadian rhythms in mammals are driven by “pacemakers” in the brain, which consist of thousands of neurons in structures called the suprachiasmatic nuclei (SCN) in the anterior hypothalamus [1] (see Figure 1). The SCN are directly connected to the retina; when this pathway is disrupted, the rest–activity cycle fails to be synchronized to the LD cycle. Normal synchronization, or entrainment, to different LD cycles is accomplished through variation in the response of the circadian pacemaker to light pulses [2],[3]. Light exposure late in the biological day, around dusk, will delay the onset of activity in a nocturnal animal, and delay the onset of inactivity in a diurnal animal. Light exposure early in the biological day (dawn) will advance the onset of activity in a diurnal species, and advance the onset of sleep in a nocturnal species. This phase-shifting effect of light is clearly a non-image forming effect of light, which depends on circadian phase and plays an important role in the temporal organization of behaviour in animals, including humans. We now know more details about the molecular machinery that gives rise to this circadian clock and how light exposure affects the expression of some of the component clock genes, and we know that this expression depends on the phase of the endogenous cycle at which light exposure occurs [4],[5].

## Light and the Circadian and Homeostatic Aspect of Sleep Regulation

Sleep is regulated by an interaction of homeostatic and circadian processes. The homeostatic mechanisms keep track of how long we have been awake and asleep, and the circadian process determines the optimal time for sleep. The homeostatic and circadian processes also influence sleep duration and the relative contribution of the two major types of sleep: non-rapid eye movement (NREM) and rapid eye movement (REM) sleep. In other words, how sleepy we are and the structure of sleep depend on how long we have been awake and the biological time of day. The SCN generate signals that influence sleepiness, sleep duration, and sleep structure. As expected, lesions of the SCN abolish the circadian aspect of sleep regulation. However, lesions of the SCN do not abolish the homeostatic aspect of sleep regulation as indexed by the deepest form of NREM sleep, which is called slow-wave sleep because during this sleep stage the electrocorticogram (ECoG) is dominated by slow wave activity (SWA) [6]. There is now a large body of evidence showing that circadian rhythmicity and homeostatic processes related to the sleep–wake cycle contribute to the regulation of many aspects of behaviour and brain function, including our performance on cognitive tasks [7].

A key question in the field concerns the effects of light on the circadian and homeostatic regulation of sleep and waking performance. Adequately timed light exposure that shifts the timing of the rhythms driven by the SCN also shifts the timing of sleep and performance rhythms. However, the effects of light on sleep–wake regulation extend beyond these phase-shifting effects. Early on, it was demonstrated that in the nocturnal rat, light can acutely suppress activity and induce sleep [8], whereas in the diurnal human, light exerts an acute alerting effect [9]. These direct effects of light on sleep–wake regulation did not receive much attention until the recent description of some of the underlying pathways and mechanisms.

## Light Input Pathways: Melanopsin, Rods, and Cones

Originally, scientists held a tacit belief that the light effects on circadian rhythms, as well as other non-image forming effects, were mediated by the classical photoreceptors that mediate vision. This view was shattered when non-image forming responses were demonstrated in mice devoid of the then known photoreceptors: light still elicited circadian phase-shifting responses [10] and the hormone melatonin was suppressed [11]. Light-induced suppression of melatonin had previously been shown to persist in some visually blind people [12]. These data, as well as the demonstration that the spectral sensitivity of non-image forming responses differed from visual responses also in humans [13],[14], were consistent with the existence of a novel photoreceptive system, subsequently identified as melanopsin. The photopigment melanopsin is expressed in the inner retina of humans [15] and other animals, and in particular in a subclass of photosensitive retinal ganglion cells (pRGCs) [16],[17]. In mice lacking the gene *Opn4*, which codes for melanopsin, phase shifts, pupillary constriction, and acute suppression of activity in response to light are all attenuated. Abolition of rods and cones, as well as the *Opn4* gene, abolishes all known image forming and non-image forming effects, demonstrating that both the classical and novel photoreceptive system contribute to these responses. Several recent, detailed studies have shown that the effects of rod and cones on non-image forming responses are transferred to the rest of the brain via the light sensitive ganglion cells [18].

The discovery of melanopsin and the pRGCs, together with the demonstration that this system is particularly sensitive to blue light, has inspired many new investigations on the effects of light on brain function and physiology [19]. For example, the combination of disrupting *Opn4* and *Rpe65* (a gene that expresses a protein involved in chromophore regeneration) can switch an animal from being nocturnal to diurnal [20]. However, the role of melanopsin in sleep regulation has not been investigated until recently.

## Role of Melanopsin in Sleep Regulation

Before the discovery of pRGCs, melanopsin, and the associated neuroanatomy, the effects of light on sleep were investigated by focusing on known visual areas in the brain, such as the visual cortex and the superior colliculus-pretectal area. Lesions in the latter area were indeed shown to greatly reduce the effects of light on sleep [21]. Recently, attention has switched to the contribution of the nonvisual system, and melanopsin in particular. It was reported that genetic ablation of *Opn4* (*Opn4^−/−^*) abolishes the light induction of sleep in mice [22]. In addition, ablation of *Opn4* abolishes the light-induced expression of *Fos*, which is an early gene and often used to identify brain structures that are activated by a stimulus, in the superior colliculus and the ventral lateral pre-optic (VLPO) area. The latter hypothalamic area is of particular interest because it receives a direct input from pRGCs [23] and plays a key role in sleep regulation [24]. Although in this experiment, light was administered only during the dark phase, i.e., when the nocturnal mouse is active, it was concluded that the direct photic mediation of sleep is predominantly mediated by OPN4. Subsequently, Altimus and colleagues investigated the effects of light on ECoG sleep in *Opn4^−/−^* mice, in mice in which the rod and cone pathways are disabled, and in mice lacking the pRGCs [25]. These authors reported that both rod/cone and melanopsin pathways contribute to light-induced sleep. The effects of light on sleep were again investigated at the beginning of the dark phase. In addition, they investigated the effects of a dark pulse at the beginning of the light phase and report that both the rod/cone and the melanopsin system contribute to the dark pulse-induced wakefulness.

## Role of Melanopsin in Sleep Regulation: Recent Advances

Although these previous studies implicated melanopsin as a mediator of the effects of light on sleep, many central questions remain unanswered. Important questions to be addressed are as follows: (1) Are all effects of light mediated by melanopsin, or do rods and cones also play a role? (2) Do these effects vary across the circadian cycle, i.e., do they differ between night and day? (3) Do these effects only change the timing of sleep, or do they also change the structure of sleep, the response to sleep loss, i.e., sleep homeostasis, and the quality of sleep and waking as assessed by ECoG oscillations?

In the current issue of *PLoS Biology*, Tsai and colleagues [26] report a detailed study on the effects of light and darkness on sleep and on brain activity in wild type and *Opn4^−/−^* mice. The study provides much additional information on the effects of light on sleep and the role of melanopsin. In contrast to most previous studies, in which the effects of light on sleep were investigated only during the biological night, Tsai and colleagues quantified these effects during both the biological night and biological day. The data show that the light-induced sleep response is abolished in *Opn4^−/−^* mice during the dark phase, but persists nearly unattenuated during the light phase. Thus, the contribution of OPN4 to sleep induction appears to be most prominent during the dark phase, whereas during the light phase rods and cones provide a substantial contribution to this effect. Another innovative aspect of this study is the detailed analysis of the effects of both light and darkness on sleep–wake behaviour. In contrast to previous studies, in which the effects of light and melanopsin, on sleep and waking were only investigated by visual scoring of ECoG, the authors applied quantitative analyses of these brain oscillations. This allowed the demonstration of effects of light and darkness and the contribution of melanopsin to the “quality” of waking. The data show that, whereas in both wild-type and *Opn4^−/−^* mice darkness induced an equivalent increase in wakefulness, the quality of this alerting effect of darkness differed between the genotypes. The alerting response, as indexed by ECoG theta and gamma oscillations, was significantly delayed in *Opn4^−/−^* mice. Another fascinating observation is that, under baseline conditions, *Opn4^−/−^* mice slept approximately 1 h less during the day (light phase). This reduction in sleep duration was primarily related to an extension of the wake bout length, i.e., wake consolidation, in *Opn4^−/−^* mice during the light phase. Thus, in the absence of melanopsin, the light-induced transition from wakefulness to sleep is delayed. Despite this increased wake duration, ECoG SWA, a marker of sleep homeostasis and depth of sleep, was significantly attenuated in *Opn4^−/−^*, in particular during the dark phase. This observation implies that lack of OPN4 may affect a fundamental aspect of sleep homeostasis, i.e., the build-up of sleep pressure during wakefulness. This interpretation was substantiated by the demonstration that the response to 6 h of sleep deprivation was also significantly attenuated in *Opn4^−/−^* mice.

## Importance and Implications of the Findings

It has been known for some time that light, circadian processes, and sleep homeostasis all contribute to sleep duration, sleep structure, and the ECoG during both sleep and wakefulness. It was thought that these processes were, to a large extent, independent and separate. However, the current study demonstrates that they interact and that alteration of light input to the brain by genetic ablation of melanopsin alters sleep duration, wake consolidation, the quality of wakefulness as quantified by ECoG, as well as sleep homeostasis. The current findings will be very useful for the development of animal models to study some of the recently described novel effects of light on brain function in humans. For example, exposure to (blue) light leads to changes in PET- or fMRI-detected brain responses to a cognitive task in humans during the night and day [27]–[29]. Blue light has been shown to be more effective than green light (the maximum sensitivity of the classical visual system) in attenuating the increase in sleepiness and deterioration of performance in the evening and during the night [30],[31]. Enriching office light with blue light leads to an increase in reported performance and a reduction in daytime sleepiness, as well as an improvement in reported night-time sleep quality [32].

These recent findings reinforce that light is not just for vision and light is not just for time keeping. Light–dark input shapes the temporal organization of behaviour through its continuous modulatory effects on brain function in interaction with the circadian timing system. This has significant implications not only for a greater understanding of these systems and how they interact, but also for our increased awareness of the importance of both natural and artificial light for human health and well being in society.
